# Inhibition of endo-lysosomal function exacerbates vascular calcification

**DOI:** 10.1038/s41598-017-17540-6

**Published:** 2018-02-21

**Authors:** Yujun Cai, Xue-Lin Wang, Alyssa M. Flores, Tonghui Lin, Raul J. Guzman

**Affiliations:** Division of Vascular and Endovascular Surgery, Department of Surgery, Beth Israel Deaconess Medical Center, Harvard Medical School, Boston, MA 02215 USA

## Abstract

Vascular calcification is a pathologic response to mineral imbalances and is prevalent in atherosclerosis, diabetes mellitus, and chronic kidney disease. When located in the media, it is highly associated with increased cardiovascular morbidity and mortality, particularly in patients on dialysis. Vascular calcification is tightly regulated and controlled by a series of endogenous factors. In the present study, we assess the effects of lysosomal and endosomal inhibition on calcification in vascular smooth muscle cells (VSMCs) and aortic rings. We observed that lysosomal function was increased in VSMCs cultured in calcification medium containing 3.5 mM inorganic phosphate (Pi) and 3 mM calcium (Ca^2+^) for 7 days. We also found that the lysosomal marker lysosome-associated membrane protein 2 was markedly increased and colocalized with osteogenic markers in calcified aortas from vitamin D_3_-treated rats. Interestingly, both the lysosomal inhibitor chloroquine and the endosomal inhibitor dynasore dose-dependently enhanced Pi + Ca^2+^-mediated VSMC calcification. Inhibition of lysosomal and endosomal function also promoted osteogenic transformation of VSMCs. Additionally, lysosome inhibition increased Pi-induced medial calcification of aortic rings *ex vivo*. These data suggest that the endosome-lysosome system may play a protective role in VSMC and medial artery calcification.

## Introduction

Medial artery calcification is prevalent in diabetes mellitus, chronic kidney disease, and with advancing age. It has been associated with morbidity and mortality related to cardiovascular diseases^1–4^. In the coronary arteries, it is strongly associated with atherosclerotic burden and independently predicts cardiovascular events^5,6^. In the lower extremities, arterial calcification predicts amputation independent of the ankle-brachial index (ABI) and atherosclerosis risk factors^7^. It is associated with increased risk of procedural complications, and it is more common in diabetic patients with foot ulcers than in those without^8^. When located in the media, arterial calcification is highly associated with increased cardiovascular morbidity and mortality, and this is particularly worse in patients with end-stage renal disease^9^.

Arterial calcification is typically divided into two major types. Intimal calcification is common in atherosclerotic plaques such as those found in the coronary and carotid arteries, whereas the medial form is independent of atherosclerosis and usually found along with elastic lamellar degradation in the muscular arteries of the leg^10,11^. Vascular calcification is known to be a tightly regulated process, controlled by a series of endogenous inhibitory factors including matrix gla protein (MGP) and pyrophosphate (PPi), and pro-osteogenic factors including Runt-related transcription factor 2 (RUNX2) and bone morphogenetic proteins (BMPs)^10,12^. More recent investigations also suggest that microRNAs^13^, endoplasmic reticulum (ER) stress^14^, the inflammasome^15^, and autophagy^16^ are involved in the regulation of vascular calcification. Vascular smooth muscle cells (VSMCs) are thought to play a central role by differentiating into osteoblast-like cells capable of releasing matrix vesicles that give rise to calcium deposition on elastin fibers^12^.

Endosomes are membrane-bound, intracellular compartments that serve to internalize and sort molecules including inorganic compounds from the plasma membrane. They subsequently fuse with lysosomes where degradation occurs^17^. Lysosomes are single membrane-enclosed organelles present in all eukaryotic cells that contain large numbers of hydrolytic enzymes. Such proteases, nucleases, and phosphatases are able to degrade extracellular and intracellular components^17,18^. Lysosomes exhibit their maximal enzymatic activity at a low pH (pH ≤ 5). The two most abundant lysosomal membrane proteins, lysosome-associated membrane protein 1 and 2, are transmembrane glycoproteins known to play a role in the protection, maintenance, and adhesion of the lysosome^19^. Lysosome-associated membrane protein 2 (LAMP2) is primarily seen in late stage endosomes and lysosomes. Lysosomal dysfunction has been associated with aging and a number of human diseases including atherosclerosis and chronic kidney disease^20^. In this study, we assess the changes in endo-lysosomal function during phosphate-induced VSMC calcification. We further evaluate the effect of endosomal and lysosomal inhibitors on phosphate stimulated VSMCs transformation and calcification of aortic rings.

## Results

### Lysosomal function is increased during VSMC calcification

**Figure 1:**
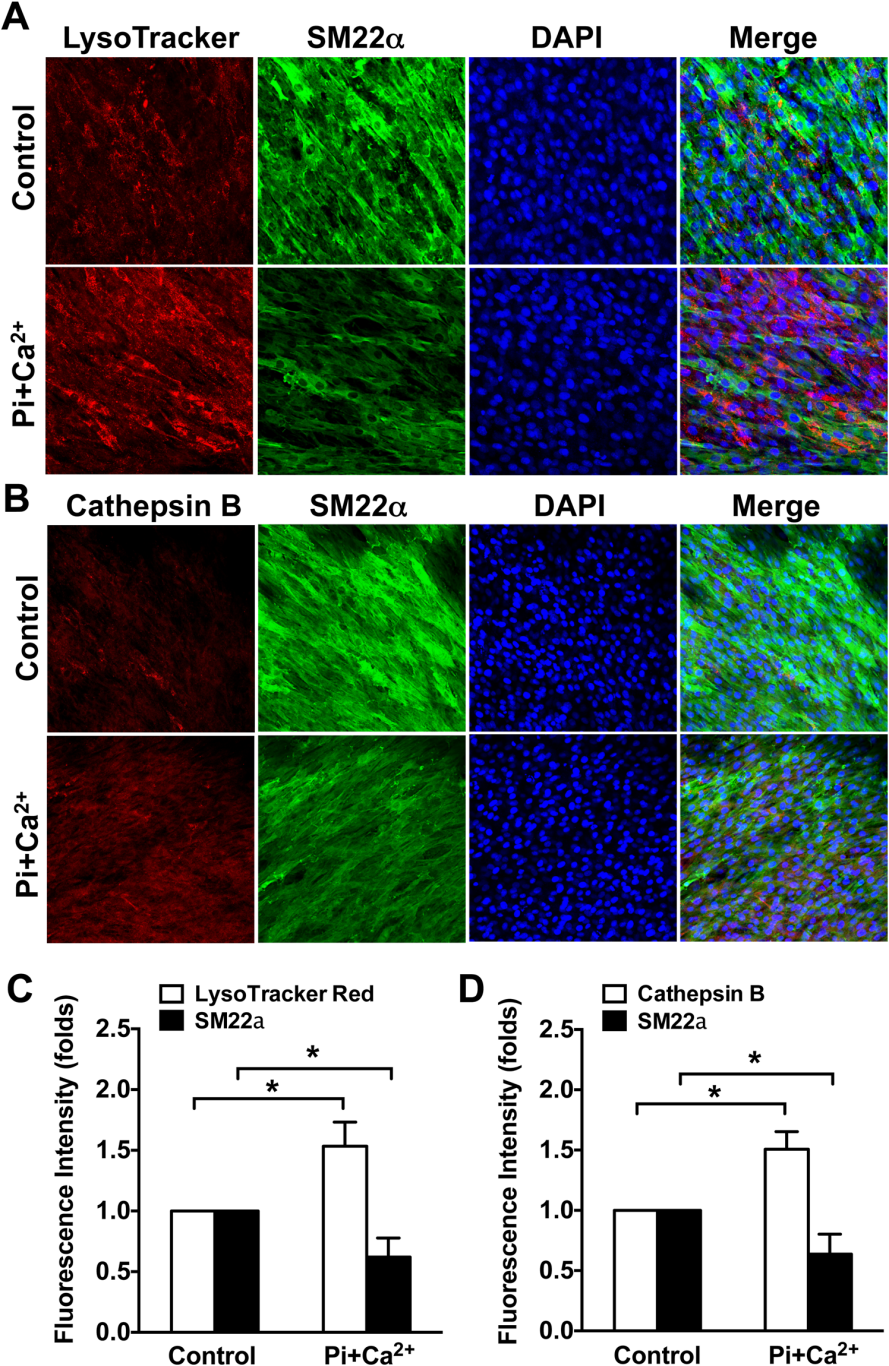
Lysosomal function is increased in calcifying VSMCs. Confluent rat aortic VSMCs were cultured on a chamber slide in a Pi + Ca^2+^ (3.5 mM phosphate/3 mM calcium) calcification medium for 7 days, the function of lysosome was evaluated by the LysoTracker red and cathepsin B magic red staining. (**A** and **C**) Representative immunofluorescent staining images and quantitative results showing increased LysoTracker intensity and decreased SM22α expression in calcifying VSMCs. (**C**–**D**) Immunofluorescent staining images and quantitative results showing effects of calcification medium on cathepsin B activity and SM22α expression. The fluorescent intensity of lysosomes was analyzed using Image J software. At least 5 fields per condition and 3 independent experiments were performed. All Values are presented as mean ± SD. *P < 0.05.

To identify whether lysosome function is altered during smooth muscle cell calcification, confluent VSMCs were cultured in medium containing 3.5 mM Pi and 3 mM Ca^2+^ for 7 days to induce calcification. LysoTracker Red and SM22 alpha (SM22α) immunofluorescence staining were used to label lysosomes and monitor VSMC phenotypic transformation. As shown in Fig. 1A and C, the VSMC contractile marker SM22α (green staining) was significantly decreased in calcifying cells compared with controls, while the intensity of LysoTracker Red (red staining) was increased, suggesting that lysosomes are increased in areas of calcifying VSMCs. Cathepsin B, a member of the lysosomal cysteine family, is mainly located within their acidic environment. It is commonly used to evaluate lysosomal function^21^. We next assessed lysosomal Cathepsin B activity using the Magic Red Cathepsin B activity assay. Consistent with our initial results, VSMCs cultured in calcification medium exhibited decreased SM22α and increased cathepsin B activity as shown in Fig. 1B and D. These results suggest that lysosomal function is increased during VSMC calcification.

### The lysosomal marker LAMP2 is upregulated in medial artery calcification *in vivo*

**Figure 2:**
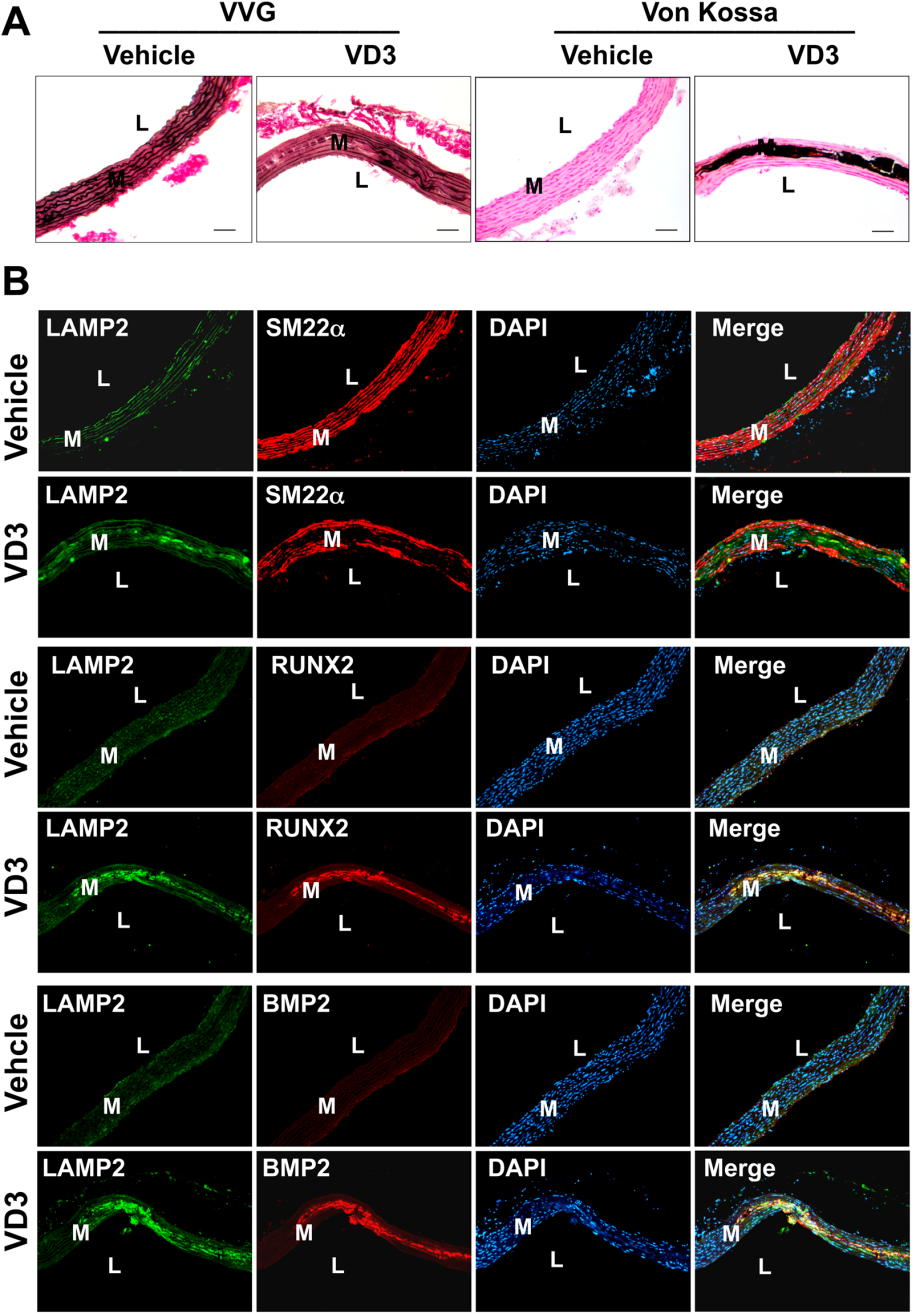
Lysosomal marker LAMP2 is increased and co-localizes with osteogenic markers in calcifying aortas. Rats were subcutaneously injected with 1.2 × 10^5^ IU/kg/day vitamin D_3_ or vehicle for 3 days and euthanized 14 days later. (**A**) Histological staining with Von Kossa and VVG showing vitamin D_3_-induced medial calcification. (**B**) Double immunofluorescent staining showing increased lysosomal marker LAMP2 fluorescence that largely overlaps with osteoblast markers RUNX2 and BMP2 in calcifying aortas. Nuclei were stained with DAPI. Bar = 50 μm. L and M refer to lumen and media, respectively.

**Figure 3:**
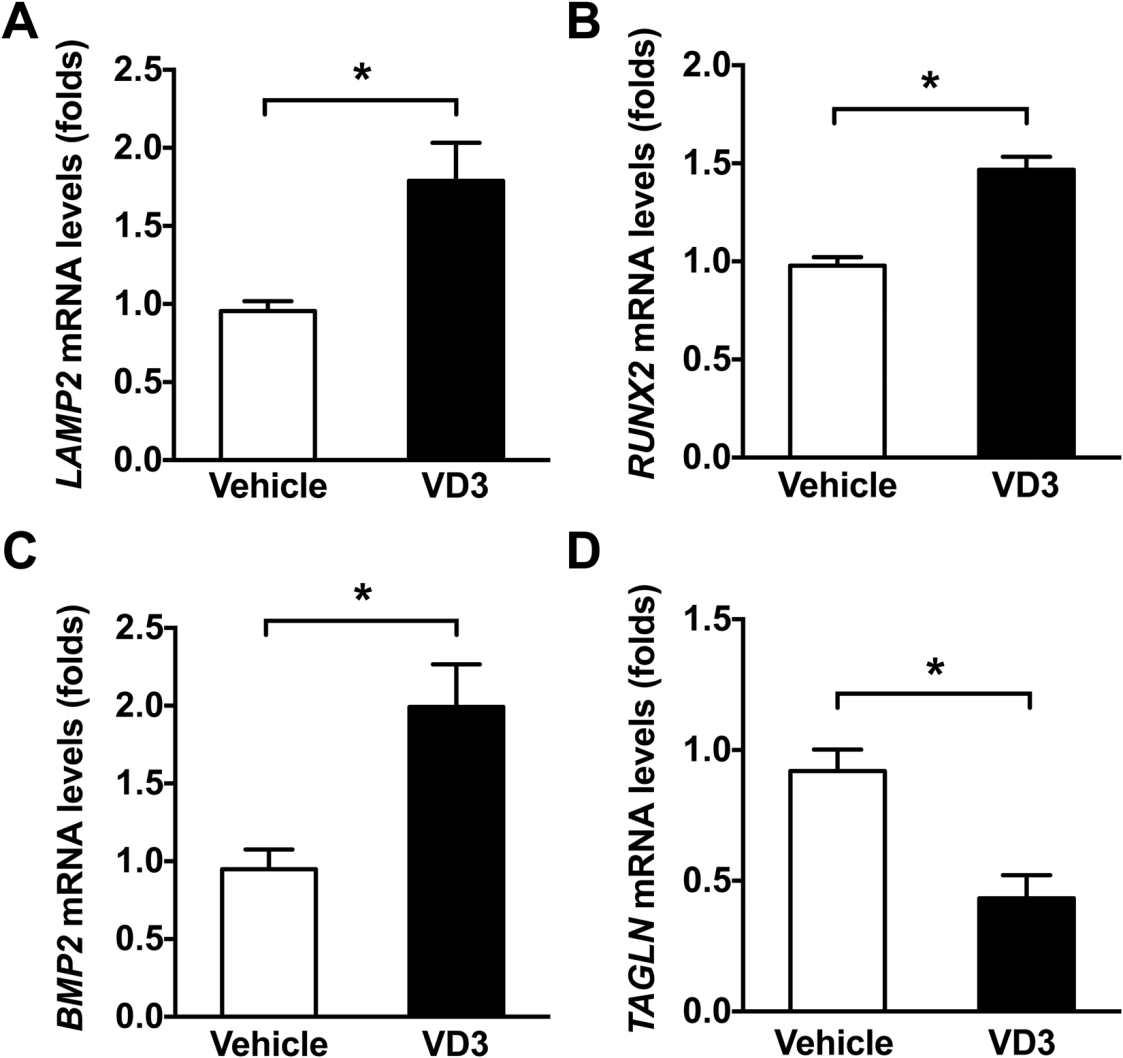
*LAMP2* is increased in calcifying rodent aortas. Rat aortic calcification was induced by subcutaneous injection with 1.2 × 10^5^ IU/kg/day vitamin D_3_ or vehicle for 3 days. After total 14 days, aortas were dissected and total RNA was isolated. qPCR results revealed that *LAMP2* was increased concomitant with osteogenic transformation showing increased *RUNX2* and *BMP2*, and decreased *TAGLN*. Six rats for each group were analyzed. Values are presented as mean ± SEM. *P < 0.05.

Lysosome associated membrane protein 2 (LAMP2) plays a pivotal role in the protection, maintenance, and signal transduction occurring within lysosomes^22^. To investigate whether lysosomal function is altered in calcifying arteries, we employed a model of medial calcification induced by vitamin D_3_^23^. As shown in Fig. 2A, subcutaneous administration of vitamin D_3_ induced profound aortic calcification that occurred in the medial layer as demonstrated by Von Kossa and Verhoeff van Giesen (VVG) staining. The osteoblast markers RUNX2 and BMP2 were increased and VSMC marker SM22α was decreased in the arterial media of vitamin D_3_-injected mice compared with vehicle control (Fig. 2B). Importantly, the lysosomal marker LAMP2 was markedly increased in calcified areas that largely overlapped with RUNX2 and BMP2 staining. In RNA isolated from vitamin D_3_ or vehicle-treated mice, quantitative PCR (qPCR) showed increased *LAMP2*, *RUNX2*, and *BMP2*, while *TAGLN* was decreased (Fig. 3). These data suggest that lysosome function is increased in pathological medial artery calcification.

### Inhibition of lysosomal function enhances VSMC calcification

**Figure 4:**
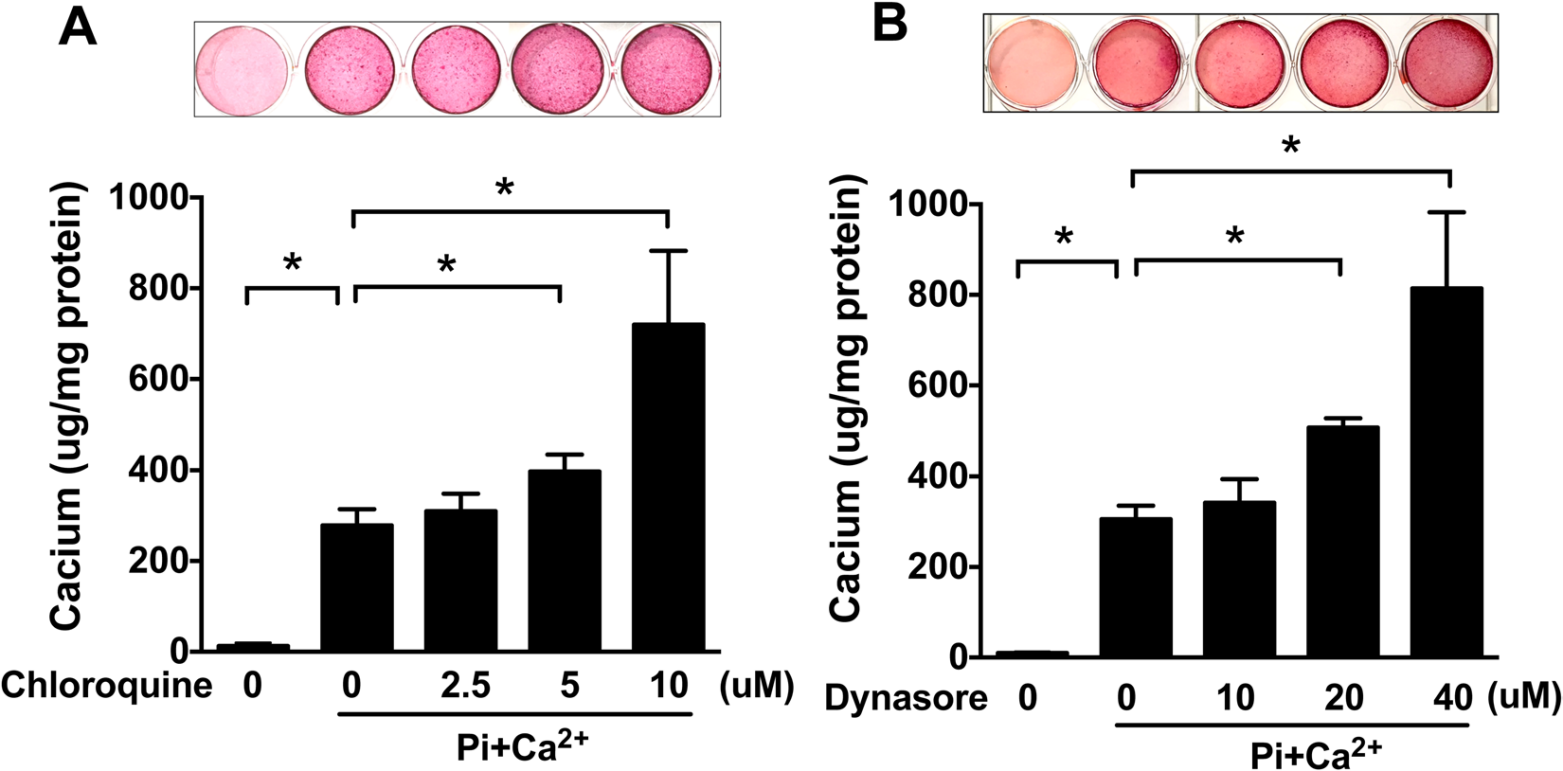
Inhibition of lysosomal and endosomal function enhances VSMC calcification. Confluent rat aortic VSMC were cultured in a Pi + Ca^2+^ (3.5 mM phosphate/3 mM calcium) calcification medium with or without indicated concentration of lysosomal inhibitor chloroquine or endosomal inhibitor dynasore for 7 days, and calcification medium was replaced every other day. (**A**) Effect of increasing concentrations of chloroquine on calcium deposition. (**B**) Effect of dynasore on calcium deposition. Top panel, Alizarin Red S staining; Bottom panel, calcium assay. The data represent 6 determinations. Values are presented as mean ± SD. *P < 0.05.

Lysosomes consist of a large number of enzymes that function in an acidic environment. Vascular calcification involves progressively enlarging deposits of calcium hydroxyapatite mineral. To study whether lysosomal function is involved in VSMC calcification, we utilized the lysosomal inhibitor chloroquine that rapidly accumulates inside the organelle, increases the pH, and prevents fusion with endosomes and autophagosomes^24,25^. As shown in Fig. 4A, when VSMCs were cultured in calcification medium for 7 days, calcium levels were significantly increased compared with normal culture medium, and this effect was further enhanced by chloroquine treatment, suggesting that increasing lysosomal function may work to suppress VSMC calcification.

### Inhibition of endosome function facilitates VSMC calcification

Accumulating evidence suggests that endocytosis plays a critical role in the early processing and degradation of extracellular materials^26^. In an attempt to determine whether endosomal function plays a role in VSMC calcification, cells were cultured in calcification medium with or without the endosomal inhibitor dynasore for 7 days. Dynasore is a cell-permeable small molecule that specifically inhibits the GTPase activity of dynamin1 and dynamin2, therefore, blocking the endocytic pathway. It is known that the GTPase dynamin is essential for clathrin-mediated endocytosis, a major process by which cells can deliver biomaterials to lysosomes^26^. As shown in Fig. 4B, when VSMCs were treated with increasing doses of dynasore, calcium accumulation was dose-dependently augmented as revealed by both Alizarin red S staining (top panel) and calcium assay (bottom panel). These data suggest that dynamin-dependent endocytosis could play a protective role in VSMC calcification.

### Inhibition of lysosomal or endosomal function augments VSMC osteogenic differentiation

**Figure 5:**
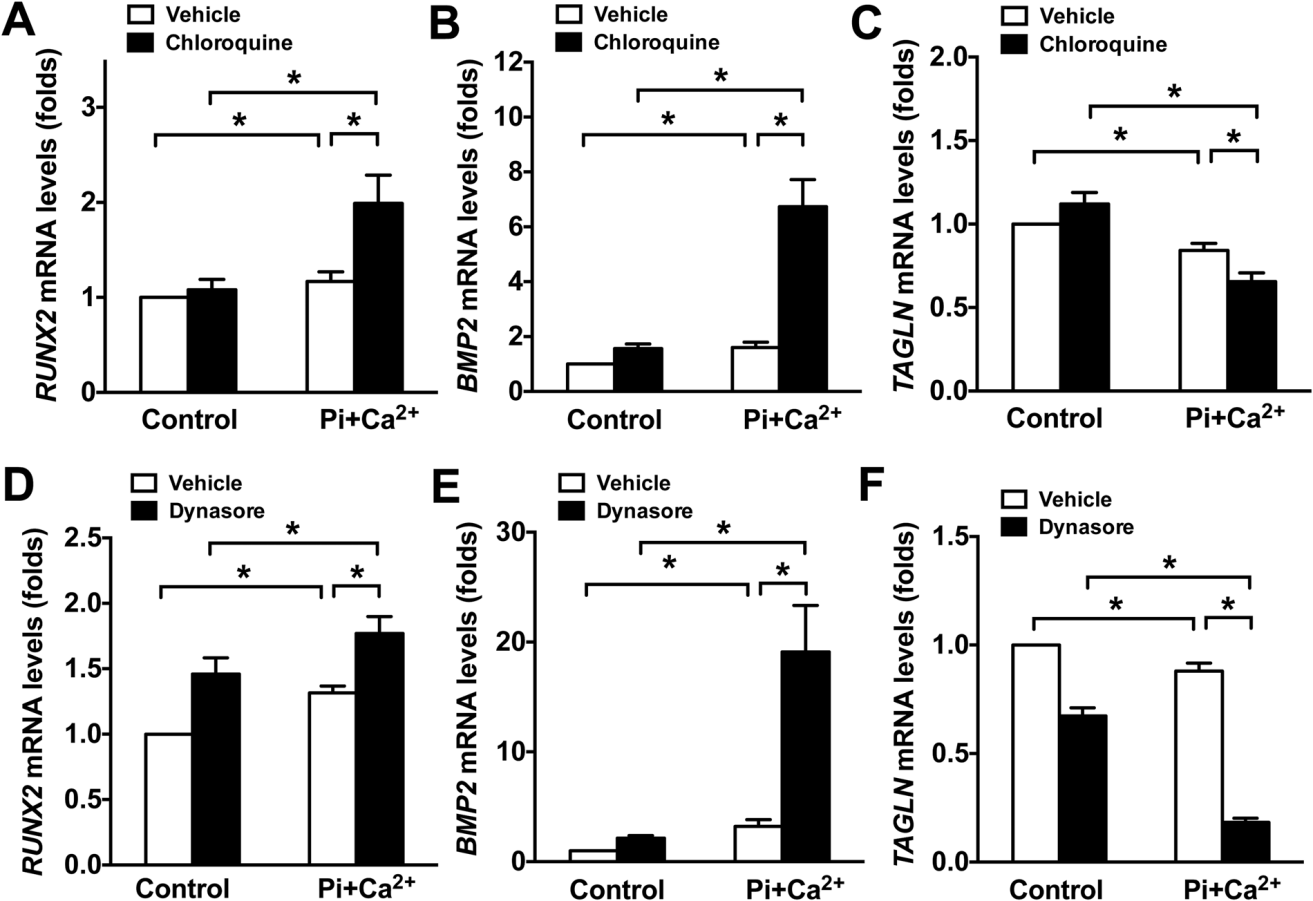
Inhibition of lysosomal or endosomal function augments osteogenic VSMC transformation. Confluent rat aortic VSMCs were cultured in Pi + Ca^2+^ (3.5 mM phosphate/3 mM calcium) calcification medium with or without 10 μM of lysosomal inhibitor chloroquine or 40 μM of endosome inhibitor dynasore for 7 days, and the fresh calcification culture medium was changed every other day. (**A**–**C**) qPCR results demonstrate effect of chloroquine on osteoblast markers *RUNX2* and *BMP2*, and VSMC contractile marker *TAGLN* in Pi + Ca^2+^-stimulated VSMCs. (**D**–**F**) qPCR results showed an enhanced effect of dynasore on Pi + Ca^2+^-induced osteogenic transformation. The data represent 6 determinations and were replicated two times. Values are presented as mean ± SD. *P < 0.05.

In response to elevated phosphate levels, VSMCs are able to undergo osteogenic transformation, a phenotypic change from a contractile to more bone cell-like state^27^. To determine whether inhibition of lysosomal or endosomal function could affect VSMC osteogenic transformation, confluent VSMCs were cultured in calcification medium with or without chloroquine or dynasore for 7 days. The osteoblast markers *RUNX2* and *BMP2*, and VSMC contractile marker *TAGLN* were assessed by qPCR. As shown in Fig. 5, *RUNX2* and *BMP2* were significantly upregulated when VSMCs were cultured in calcification medium. However, this effect was markedly enhanced by chloroquine treatment (Fig. 5A–B). Conversely, *TAGLN* was significantly decreased in calcification medium compared with normal culture medium, and it was further reduced by chloroquine treatment (Fig. 5C). In line with this, treatment with dynasore also significantly enhanced the effects of Pi + Ca^2+^ on VSMC osteogenic transformation (Fig. 5D–F). Interestingly, Dynasore treatment alone was also able to increase *RUNX2* and decrease *TAGLN*, suggesting that endosomal inhibition affects osteogenic transformation. Collectively, these data suggest that the endosome/lysosome system plays a pivotal role in protecting VSMCs from Pi + Ca^2+^-mediated osteogenic transformation.

### Lysosomal inhibition enhances medial artery calcification

**Figure 6:**
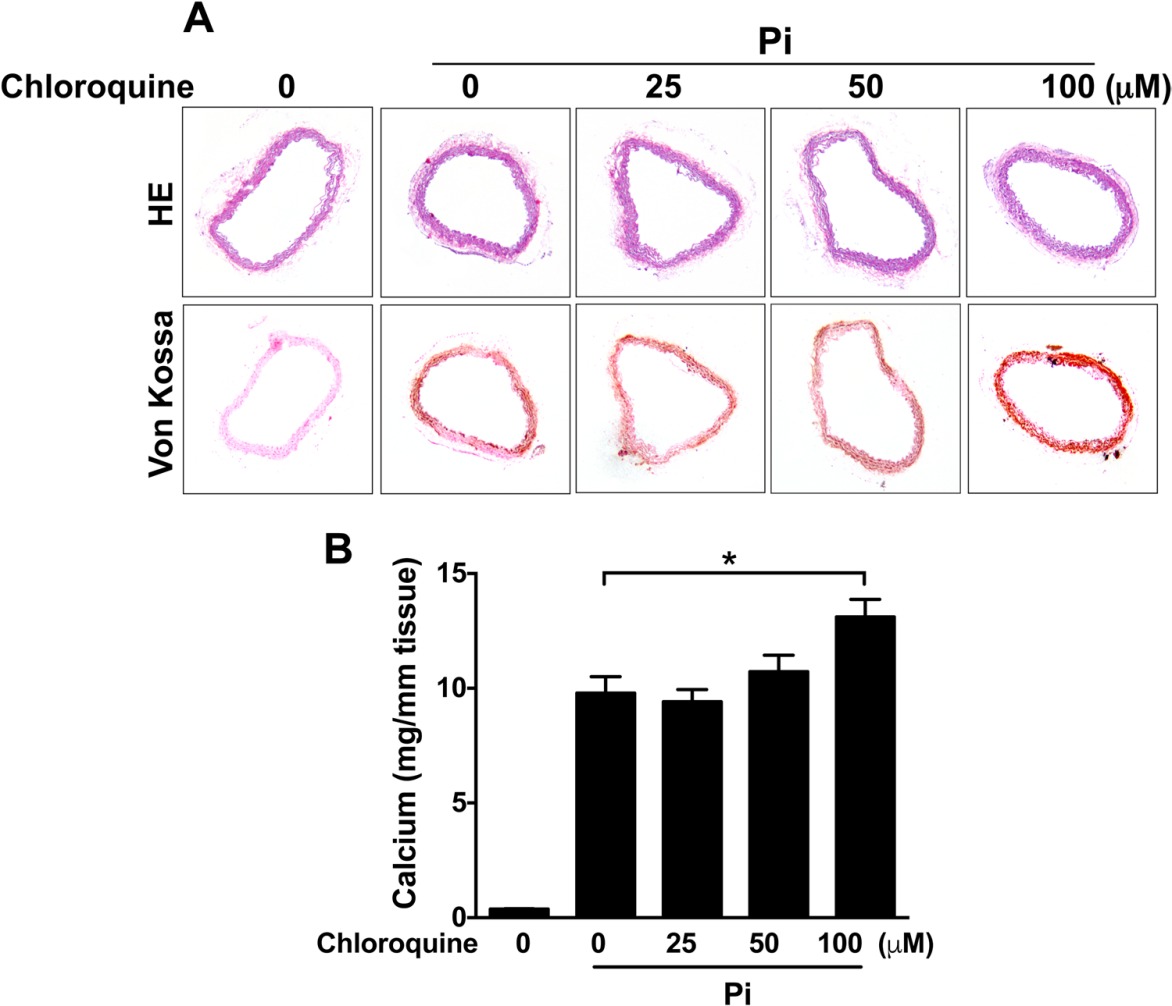
Lysosomal inhibition increases medial calcification. Mouse thoracic aortas were isolated from C57BL/6 J mice and cut into 3 mm-long aortic rings. Aorta segments were cultured in calcification media (DMEM + 10% FBS + 2.6 mM Pi) with or without chloroquine for 8 days. (**A**) HE and Von Kossa staining showing chloroquine effect on Pi-induced medial calcification. (**B**) Calcium assay showing effect of chlorquine on Pi-induced calcium accumulation. The data represent 6 aortic rings per each group. Values are presented as mean ± SEM. *P < 0.05.

We next used an organ culture model of medial calcification. Aortic rings incubated in calcification medium containing elevated phosphate levels for 8 days develop medial artery calcification similar to that seen *in vivo*^28^. Calcium levels were significantly increased by chloroquine treatment in Pi-stimulated aortic rings (Fig. 6B). Von Kossa staining also showed that addition of chloroquine further increased calcium deposits in the medial layer of aortas (Fig. 6A). These data suggest a role for endo-lysosomal function in protecting arteries from medial calcification.

## Discussion

Medial artery calcification is a pathological response to metabolic derangements most commonly seen in diabetes and renal failure. It is associated with increased morbidity and mortality related to cardiovascular diseases and predicts outcomes in patients with peripheral artery disease such as amputation and death^3,29^. Currently, there are no therapeutic agents available to prevent arterial calcification or slow its development. Medial VSMCs play a central role in vascular calcification as they can transform into more osteogenic cell types in response to elevated phosphate levels^12^. In this study, we observed that endo-lysosomal function is increased in calcifying VSMCs *in vitro* and aortic segments *in vivo* as demonstrated by Lysotracker and LAMP2 staining. Inhibition of endosome function by dynasore, and lysosome function by chloroquine significantly increased VSMC calcification and osteogenic transformation. These data suggest that endo-lysosomal function plays an important regulatory role in vascular calcification and that targeting these processes may provide a new strategy to suppress arterial calcification and improve outcomes in patients with arterial disease.

Endosomes are membrane-bound compartments that internalize molecules from the extracellular space and plasma membrane. They provide an environment where internalized materials can be sorted before they are degraded in the lysosomes. Three separate pathways are known to be involved in endocytosis. Clathrin-mediated endocytosis is the most common, while the caveolae and non-clathrin/non-caveolae mediated pathways follow^26^. We used dynasore, a cell-permeable, reversible, and non-competitive inhibitor of dyanamin 1 2 GTPase, to inhibit clathrin-mediated endosomal function. We found that dynasore dose-dependently increased calcification of VSMC, suggesting that clathrin-mediated endocytosis is involved in protecting against vascular calcification. Future studies on the role of endosomal pathways in calcification are needed.

Lysosomes play a critical role in cellular homeostasis and are implicated in a wide array of physiological processes including cellular clearance, lipid and energy metabolism, and bone remodeling. The autophagy/lysosome pathway is used to degrade intracellular macromolecules and dysfunctional organelles^30^, whereas the endosome/lysosome pathway is mainly involved in degrading materials that are brought in from the extracellular environment^31,32^. Lysosomes contain over 60 different hydrolytic enzymes that function in an acidic milieu. Chloroquine has been shown to inhibit endosomal and lysosomal function by preventing acidification required for enzymatic activity. Recent studies suggest that chloroquine may also function by inhibiting the fusion of autophagosomes with lysosomes through a pH-dependent mechanism^33^. In the present study, we observed that chloroquine treatment enhanced calcification and osteogenic transformation in cultured VSMCs. It also increased medial calcification in aortic rings cultured in medium with elevated phosphate levels, suggesting that lysosomal function play a role in preventing vascular calcification.

Bone morphogenetic proteins (BMPs) are part of the TGF-beta superfamily of signaling proteins that play critical roles in determining cell fate and function^34^. BMP2 is highly expressed in calcifying arteries and increasing VSMC-specific BMP2 expression accelerates vascular calcification. Others groups and we ourselves have previously shown that BMP2 is involved in VSMC calcification^35,36^. Interestingly, in the present study, we observed that chloroquine and dynasore dramatically increase BMP2 expression and provoked a synergistic effect, suggesting that BMP2 could be a major target for endosome and lysosome-regulated vascular calcification.

It has been shown that high phosphate levels are capable of promoting autophagy in VSMCs as measured by staining for the autophagosome marker microtubule-associated protein 1A/1B light chain 3B-II (LC3-II), and inhibition of autophagy increased VSMC calcification as well as aortic calcification in an organ culture model^16,37^. In the present study, we observed that inhibition of endo-lysosomal function significantly enhanced VSMC calcification. These findings suggest that both endosomal and autophagosomal pathways are involved in the control of VSMC phenotype and vascular calcification. Interestingly, vascular cells exposed to basic calcium phosphate (BCP) crystals exhibited increased intracellular calcium levels and cell death. This was reversed by lysosomal de-acidification with bafilomycin. When applied to VSMCs, BCP crystals caused release of high levels of calcium in lysosome acidic milieu. However cell death did not occur when BCPs were applied in serum-containing medium suggesting that protein crystal aggregates are not as toxic^38^. It also has been demonstrated that high Pi could promote calcification by stimulating VSMC apoptosis^39^. In uremic mice and dialysis patients, extensive VSMC apoptosis is evident^40,41^. Moreover, chloroquine has also been shown to mediate cell death by disrupting lysosome function in various cell types^42,43^. Importantly, a recent study has shown that 7-ketocholesterol, a major component of oxidized low-density lipoprotein caused lysosome dysfunction and in turn exacerbated VSMC calcification^44^. Therefore, it is possible that chloroquine enhances vascular calcification by disrupting lysosomal function and subsequently inducing cell death.

**Figure 7:**
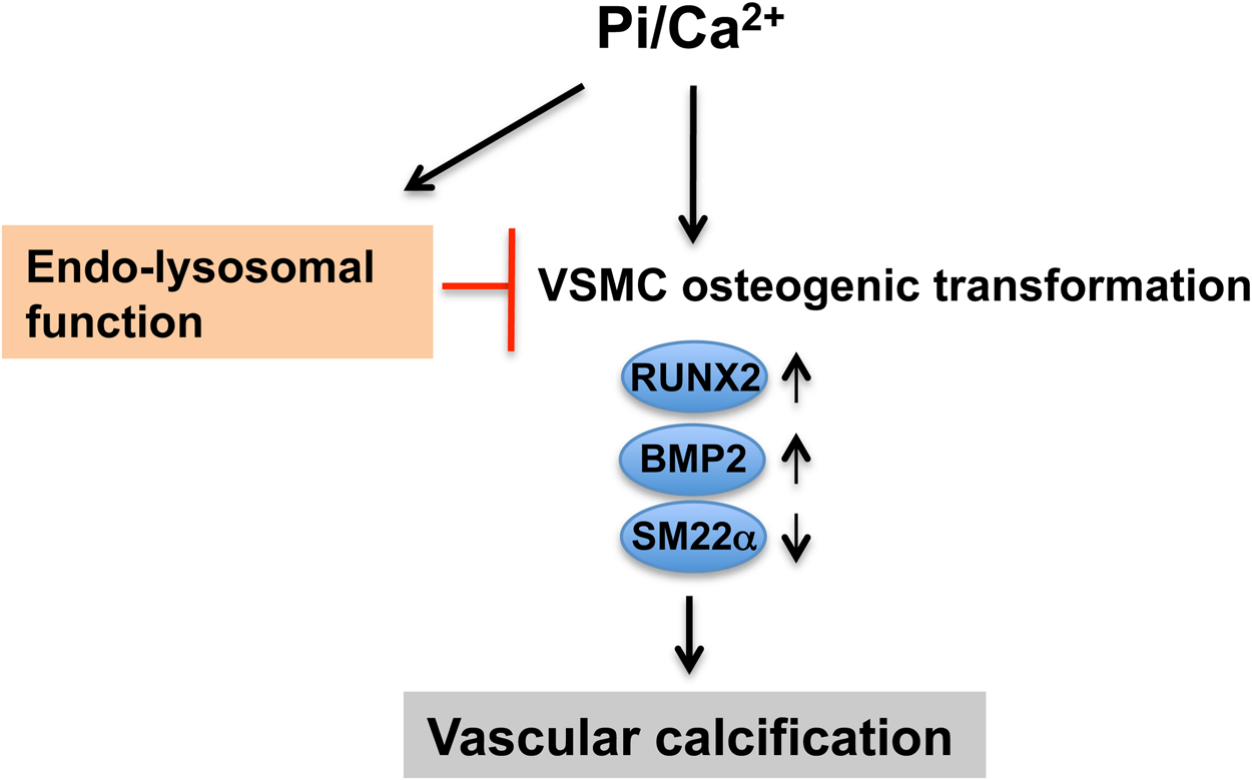
Model of endo-lysosome regulating vascular calcification in VSMCs. Elevated Pi/Ca^2+^ can increase endo-lysosomal function, which is capable of partially reducing Pi/Ca^2+^-induced VSMC osteogenic transformation and subsequent calcification.

In summary, we have demonstrated that inhibiting endosomal and lysosomal function can increase smooth muscle cell osteogenic transformation and calcification (Fig. 7). These findings suggest that preserving endo-lysosomal function may provide a new strategy to suppress medial artery calcification in patients with vascular disease.

## Methods

### Reagents

Cholecalciferol (vitamin D_3_), sodium monohydrogen phosphate heptahydrate (Na_2_HPO_4_.7H_2_O), sodium dihydrogen phosphate monohydrate (NaH_2_PO_4_.H_2_O), calcium chloride (CaCl_2_), chloroquine diphosphate, dynasore hydrate, Alizarin Red S, Silver nitrate, and nuclear fast red were purchased from Sigma-Aldrich (St. Louis, MO). Dulbecco’s Modified Eagle’s Medium (DMEM) and fetal bovine serum (FBS) were obtained from Thermo Fisher Scientific. Vitamin D_3_ was dissolved in ethanol/kolliphor EL/dextrose prior to subcutaneous injection.

### Animals

All animal usage was reviewed and approved by Institutional Animal Care and Use Committee (IACUC) at Beth Israel Deaconess Medical Center (086–2014 and 093–2016). All animal experiments and procedures were performed in accordance with the approved guidelines. Sprague-Dawley rats were purchased from Charles River Laboratories and C57BL/6J mice were obtained from Jackson Lab Laboratories. Animals were fed with a normal rodent diet ad libitum.

### Rat aortic VSMC culture and calcification induction

VSMCs were prepared using enzymatic digestion of aortas from male Sprague-Dawley rats (250 ± 50 g) as previously described^45^. Cells were grown in culture medium (DMEM + 10% FBS + 1% pen/strep) in a humidified incubator (37 °C, 5% CO_2_), and used for the experiments from passages 7 to 12. To induce calcification, the final concentrations of inorganic phosphate (Pi) and calcium (as CaCl_2_) were increased to 3.5 mM and 3 mM respectively. Cells were incubated in calcification medium for 7 days and the medium was replaced every other day.

### Medial artery calcification rat model

To induce medial artery calcification, male Sprague-Dawley rats (250 ± 50 g) were subcutaneously injected with 1.2 × 10^5^ IU/kg/day vitamin D_3_ (dissolved in denatured alcohol/kolliphor/dextrose) for 3 consecutive days as described previously^23^. After 14 days, animals were perfused with saline, and the thoracic aortas were dissected for qPCR. The aortas were fixed with 10% neutral buffer formalin (10% NBF) and embedded in paraffin for histology and immunostaining.

### Aortic ring organ *ex vivo* culture

Aortic ring organ culture was performed as previously described^28^. Thoracic aortas were isolated from 8 week old C57BL/6 J mice, gently cleared of surrounding tissues and cut into 3 mm-long segments. The aortic segments were incubated in a calcification medium (DMEM + 10% FBS + 1% pen/strep + 2.6 mM Pi) for 8 days at 37 °C in a humidified 5% CO_2_ incubator. Medium was replaced every other day.

### Calcification assessment

Calcium content was determined as previously described^23,36^. At the end of the experiment, VSMCs or aortic organ rings were washed with saline and decalcified in 200 μl 0.6 N HCl for 24 h. Calcium levels were measured using the o-cresolphthalein complexone method. Protein concentration was measured using a Pierce™ BCA Protein Assay Kit (Thermo Fisher Scientific). Results were expressed as µg/mg protein for VSMCs and mg/mm for aortic rings. VSMCs were also fixed with 4% Paraformaldehyde (4% PFA), and stained with 2% Alizarin Red S Solution to visualize the extent of calcification^36^. Additionally, aorta segments were fixed with 10% NBF and embedded in paraffin. Cross-sections were used for staining with Von Kossa’s and Verhoeff-Van Gieson’s (VVG) methods to evaluate the medial calcification.

### RNA isolation and Quantitative PCR

Total RNA was isolated from VSMCs or rat aortas using a RNeasy Mini Kit (Qiagen). 1 ug total RNA was utilized to synthesize cDNA using an iScript cDNA Synthesis kit (Bio-Rad). The cDNA equivalent to 10 ng total RNA was applied as template in qPCR reaction using the PowerUp™ SYBR® Green Master Mix (Thermo Fisher Scientific). The amplifications were performed using an AB 7500 Fast Real-Time PCR System (Applied Biosystems). The melt curve was also performed and analyzed to avoid any contaminations. The relative mRNA levels were obtained using the comparative Ct method and normalized with glyceraldehyde-3-phosphate dehydrogenase (GAPDH). The primers used in qPCR are LAMP2 (Forward: 5′-AGCAGTTGTGGCGATGATAAG-3′; Reverse: 5′- CTGAGATGCTTCCTTGGTGAAA-3′), RUNX2 (Forward: 5′-GCCACTTACCACAGAGCTATTA-3′; Reverse: 5′-GGCGGTCAGAGAACAAACTA-3′), BMP2 (Forward: 5′-TGTGAGGATTAGCAGGTCTTTG-3′; Reverse: 5′-TTGTGGAGTGGATGTCCTTTAC-3′), SM22a (Forward: 5′-AGAGGACTGTAATGGCTTTGG-3′; Reverse: 5′-CTGTCTGTGAACTCCCTCTTATG-3′), and GAPDH (Forward: 5′-GATGCTGGTGCTGAGTATGT-3′; Reverse: 5′-GCGGAGATGATGACCCTTT-3′).

### Immunofluorescent staining

Aortas isolated from vehicle or vitamin D_3_-treated rats were fixed with 10% NBF and embedded in paraffin. The sections were deparaffinized and treated with 10 mM HIER citrate buffer (pH 6.0) for antigen retrieval, and then blocked with Dako serum-free blocking solution (Dako), followed by incubation of secondary antibody Alexa Fluor 488 or 546 (Thermo Fisher Scientific). The antibodies including anti-LAMP2 (Santa Cruz Biotechnology, sc-20004), anti-RUNX2 (BML Life science, D130-3), anti-BMP2 (Abcam, ab14933), and anti-SM22α (Abcam, ab14106) were used. Nuclei were stained with DAPI. Cells were visualized with Zeiss AxioImager M1 microscope.

### Lysosomal function analysis

Lysotracker red DND-99 (Thermo Fisher Scientific), a fluorescent acid tropic probe for labeling and tracking acidic organelles in live cells, was used to visualize lysosomes^46^. Confluent VSMCs were treated with the calcification culture medium for 7 days, and then added with 1 μM Lysotracker red, followed by culture for 2 h. Moreover, Magic Red Cathepsin B (ImmunoChemistry Technologies) was also used to examine lysosomal function. Confluent VSMCs were treated with the calcification culture medium for 7 days, and then added with cathepsin B substrate reagent, MR-(RR)_2_, followed by incubation for 1 h. VSMCs were fixed with 4% PFA and stained with anti-SM22α antibody. Nuclei were stained with DAPI. Cells were visualized and images were captured using with Zeiss LSM 880 Confocal microscope. To quantify the function of lysosomes, the fluorescent intensity of lysosomes in each field was analyzed using Image J software. At least 5 fields per condition were measured.

### Statistical analysis

Statistical analyses were performed using GraphPad Prism 6.0 software. Differences were analyzed by one-way ANOVA. *P* values less than 0.05 were considered significant.
